# Costs and benefits of natural transformation in *Acinetobacter baylyi*

**DOI:** 10.1186/s12866-017-0953-2

**Published:** 2017-02-15

**Authors:** Nils Hülter, Vidar Sørum, Kristina Borch-Pedersen, Mikkel M. Liljegren, Ane L. G. Utnes, Raul Primicerio, Klaus Harms, Pål J. Johnsen

**Affiliations:** 1 Genomic Microbiology, Institute of Microbiology, Christian-Albrechts-University Kiel, Am Botanischen Garten 11, 24118 Kiel, Germany; 2 Department of Pharmacy, Faculty of Health Sciences, UiT The Arctic University of Norway, P.O. Box 6050 Langnes, Tromsø, Norway; 3 Department of Food Safety and Infection Biology, Faculty of Veterinary Medicine and Biosciences, Norwegian University of Life Sciences, P.O. Box 8146 Dep, 0033 Oslo, Norway; 4 Centre for Ecolgical and Evolutionary Synthesis, Faculty of Mathematics and Natural Sciences, University of Oslo, P.O. Box 1066 Blindern, 0316 Oslo, Norway; 5 Centre for GeoGenetics, Natural History Museum of Denmark, University of Copenhagen, Øster Voldgade 5-7, 1350 Copenhagen K, Denmark

**Keywords:** Competence, Natural transformation, Horizontal gene transfer, Bacterial evolution, DNA repair, DprA

## Abstract

**Background:**

Natural transformation enables acquisition of adaptive traits and drives genome evolution in prokaryotes. Yet, the selective forces responsible for the evolution and maintenance of natural transformation remain elusive since taken-up DNA has also been hypothesized to provide benefits such as nutrients or templates for DNA repair to individual cells.

**Results:**

We investigated the immediate effects of DNA uptake and recombination on the naturally competent bacterium *Acinetobacter baylyi* in both benign and genotoxic conditions. In head-to-head competition experiments between DNA uptake-proficient and -deficient strains, we observed a fitness benefit of DNA uptake independent of UV stress. This benefit was found with both homologous and heterologous DNA and was independent of recombination. Recombination with taken-up DNA reduced survival of transformed cells with increasing levels of UV-stress through interference with nucleotide excision repair, suggesting that DNA strand breaks occur during recombination attempts with taken-up DNA. Consistent with this, we show that absence of RecBCD and RecFOR recombinational DNA repair pathways strongly decrease natural transformation.

**Conclusions:**

Our data show a physiological benefit of DNA uptake unrelated to recombination. In contrast, recombination during transformation is a strand break inducing process that represents a previously unrecognized cost of natural transformation.

**Electronic supplementary material:**

The online version of this article (doi:10.1186/s12866-017-0953-2) contains supplementary material, which is available to authorized users.

## Background

Naturally transformable bacteria take up DNA from the environment upon entering a physiological state called competence [1, 2]. If sufficient homology is present in the taken-up DNA it may be integrated in the recipient genome and in turn combine beneficial alleles in the same genetic background, provide novel traits, and remove deleterious mutations [2–6]. Conversely, natural transformation may also cause harmful changes such as acquisition of deleterious mutations present in external DNA [7]. It is clear that natural transformation shapes bacterial genomes, and the mechanistic insights to DNA uptake and integration is well described for a number of bacterial species, reviewed in [2]. Yet, the selective forces responsible for the evolution and maintenance of natural transformation are still debated [6, 8, 9].

Natural transformation is considered a driving force of bacterial evolution [2, 10, 11], and mathematical models support that natural competence has evolved and is maintained as a mechanism for genome plasticity that lead to increased environmental adaption rates [12, 13]. In contrast, natural transformation has also been hypothesised to support genome stability [14–16] and to remove deleterious mobile genetic elements from chromosomes [17]. Experimental evolution approaches using various bacterial species competent for natural transformation including *Helicobacter pylori*, *Acinetobacter baylyi* and *Streptococcus pneumoniae* have revealed that the impact of natural transformation on rates of adaptive evolution can be beneficial [18, 19], neutral [20], and/or context dependent [21, 22]. Taken together, these reports suggest that the widely accepted idea that natural transformation accelerates bacterial adaptation lacks generality.

Although natural transformation contributes to genome evolution [2], the immediate effects of DNA uptake and recombination in individual cells remain unclear and several non-mutually exclusive hypotheses have been proposed. One hypothesis suggests that taken-up DNA may be used as a source of phosphate, nitrogen, carbon, or nucleotides [8, 23, 24]. Observations that expression of competence genes is controlled by carbon catabolite repression in *Escherichia coli* [25], *Haemophilus influenzae* [25, 26]*, Vibrio cholerae* [27] and *Streptococcus gordonii* [28] support this hypothesis. More specific links between intracellular availability of nucleotides, their precursors and natural competence have been found in *H. influenzae* where purine depletion activates the competence activator *sxy* [23], and in *Vibrio cholerae* competence is repressed by exogenous levels of cytidine [29].

An alternative hypothesis suggests that taken-up DNA can be used as template for repair of genomic DNA damages [9, 30]. Four reports by Michod and co-workers provided experimental evidence in favour of this explanation in *Bacillus subtilis* [9, 31–33]. Increased transformation rates were observed when homologous DNA, but not heterologous DNA, was added after exposure of cells to UV-light [33]. However, other reports in *B. subtilis* and *H. influenzae* [34, 35], as well as in *S. pneumoniae* [36, 37] rather suggest that competence induction may be a general stress response. This is further supported by observations that competence-specific transcriptional regulators in *S. peumoniae* and *B. subtilis* control genes whose functions are linked to stress response (reviewed in [2]). The induction of competence by DNA damaging agents in *S. pneumoniae* [38], *Legionella pneumophila* [39] and *H. pylori* [40] also favour the “DNA-repair hypothesis” for the evolution and maintenance of natural transformation*.*

In the genus *Acinetobacter* competence is not regulated by DNA damages [41, 42] and in *A. baylyi* maximum competence is reached during exponential growth-phase [43]. Under optimal conditions up to 25% of all cells in a competent *A. baylyi* culture are transformed [44]. During transformation competent cells bind extracellular double-stranded DNA [4, 45] and a single-stranded DNA fragment is transported into the cytoplasm [1, 4]. While some bacterial species e.g., *H. influenzae* and species in the family of *Neisseriaceae* selectively take up isogenic DNA [46, 47], other species such as *B. subtilis* and *A. baylyi* transfer DNA of any source into the cytoplasm [44, 48]. Homologous recombination is then initiated by DprA-mediated loading of RecA onto the single-stranded DNA [49, 50] and by subsequent RecA-mediated strand invasion and integration of the donor DNA into the recipient genome. Previous reports on *A. baylyi* indicate that genomic integration frequently cause single-strand breaks in the transforming cell that can turn into double-strand breaks when unrepaired [51, 52].

In this study, we used *A. baylyi* to determine the immediate benefits and costs of DNA-uptake and -integration under both benign and stressful conditions. Our data show that uptake of both homologous and heterologous DNA increased fitness of competent *A. baylyi* cells. The fitness increment was independent of DNA damage and did not involve genomic integration of the acquired DNA. We further show that genomic integration of homologous DNA both reduced transformant fitness when exposed to UV-induced stress, and under benign conditions strongly depended on the RecBCD and RecFOR DNA strand break repair pathways.

## Methods

### Strains, media and DNA

The strains used in this study (Additional file 1: Table S1) are derivatives of *Acinetobacter baylyi* strain JV28 [53] and were constructed using standard molecular biology techniques [54]. Plasmids and primers used in this study are listed in supplementary tables (Additional file 1: Table S2 and Table S3). JV28 is derived from *A. baylyi* ADP1 (GenBank NC_005966) [55]. Strain LCQ2 was constructed by crossing the *trpE* ^*+*^ wildtype allele into JV28 as described [56]. KOM130 was constructed by replacing the *comB-F* operon of JV28 with the Δ*comB-F*::*dhfr1* allele [20, 22] by natural transformation with DNA isolated from strain ADP1200Com- [22]. Cotransformation of unwanted markers was excluded phenotypically (TrpE^−^) or by PCR (*lifO-lipB*::*aphA3*′). The *dprA* gene (ACIAD0209) of *A. baylyi* was inactivated by replacing an internal 349-bp fragment of *dprA* with an *aacC1* (gentamicin resistance) gene using Splicing by Overlap Extension PCR [57]; see Additional file 2 for details]. The ∆*uvrA (*ACIAD3455), ∆*recF* (ACIAD0003) and ∆*recR* (ACIAD2312) mutations were constructed using the plasmid pGT41 as described [58, 59], see Additional file 2. The Δ*uvrA* strain KOM141 was obtained by replacing *uvrA* ^+^ of JV28 by the Δ*uvrA*::(*nptII sacB*) allele, and subsequent replacement of Δ*uvrA*::(*nptII sacB*) by the Δ*uvrA* allele. The *recO* (ACIAD2578) deletion allele was constructed similarly using a modified pGT41 plasmid (pGT33), and the plasmids containing the Δ*recO*::(*nptII sacB*) and Δ*recO* alleles were provided by J. de Vries (University of Oldenburg, Germany). Similar to Δ*uvrA*, the ∆*recF*, ∆*recR* and ∆*recO* alleles were each crossed into the JV28 strain, giving strains MKD1, MKD2 and KOM82, respectively. The ∆*recR* and ∆*recF* alleles were subsequently inserted into the KOM82 mutant, resulting in strain MKD3 (∆*recFOR*). Finally, the ∆*recBCD* allele [52] was crossed into each of the MKD3, MKD1, KOM82 and MKD2 mutants, giving the strains MKD6, MKD4, KOM86 and MKD5, respectively. All strains were verified phenotypically (sensitivity to UV irradiation; transformation by 2 kbp donor DNA molecules) and by PCR. The donor DNA plasmid pSBP1 was constructed by inserting a PCR product of the *lifO-lipB*::*aphA3* insertion allele [60] (amplified with ACIAD3308-f/ACIAD3309-down) of ADP1200Com + ^KanR^ [22] into the *Hin*cII site of the pUC19 plasmid vector conferring kanamycin resistance.

Bacterial cultures were incubated at 30 °C in Luria Bertani broth (LB) (BD Difco™, USA). Transformants were scored on LB agar plates supplemented with kanamycin (Sigma-Aldrich, Germany) at 50 mg l^−1^ or 10 mg l^−1^ (plating efficiencies were indistinguishable), and trimethoprim at 250 mg l^−1^.

Genomic and plasmid DNA was isolated using QIAGEN Genomic-tip (QIAGEN, Germany) and the QIAGEN genomic DNA or plasmid DNA purification kit, respectively, according to the manufacturer’s instructions.

### Pairwise competitions

Overnight cultures of a transformation-proficient test strain (LCQ2 or NH29) and the DNA uptake-deficient competitor KOM130 were grown in ten ml for 16 h at 30 °C. The cells were pelleted, re-suspended and diluted 100-fold (approximately 10^7^ cells ml^−1^) in PBS [54] pre-warmed to 30 °C. One ml aliquots of each suspension were mixed, transferred to a sterile Petri dish and irradiated with ultraviolet (UV) light using a germicidal lamp. One ml of the irradiated cells was transferred to tubes containing one ml of pre-warmed double-strength LB (2 × LB) with genomic DNA [isogenic (ADP1200Com + ^KanR^) or heterologous (salmon sperm); 2 μg ml^−1^] or with DNase 1 (100 μg ml^−1^) and aerated in the dark for 24 h at 30 °C. The cells were plated in appropriate dilution on LB medium with and without trimethoprim (250 mg l^−1^; in some control experiments: 10 mg l^−1^ kanamycin) after two and 24 h. The plates were incubated at 30 °C for 24 h, colonies were counted, and the titers at both time points for the test strain (T_t-2_ and T _t-24_) and the competitor strain (C _t-2_ and C _t-24_) were calculated. The relative fitness *w* of the test strain was calculated as *w* = ln(T _t-24_/T _t-2_)/ln(C _t-24_/C _t-2_).

### UV survival measurements

Overnight cultures of *A. baylyi* were treated as described for the pairwise competitions but without mixing with a competitor and irradiated using a germicidal lamp. Control assays were treated accordingly without irradiation (UV_0_). One ml of the cells was then mixed with one ml pre-warmed 2 × LB, amended with pSBP1 plasmid DNA (0.1 μg ml^−1^; 1800 molecules per cell) linearized with *XmnI* (New England Biolabs) or with homologous genomic DNA from strain ADP1200Com + ^KanR^ (2 μg ml^−1^; 50 genome equivalents per cell), and aerated without light for two hours before DNase 1 (100 μg ml^−1^) was added. pSBP1 is pUC19 with a two kbp insert from the *A. baylyi* chromosome with a Kan^R^ marker (*aphA3*) cloned in the centre of the insert (Additional file 1: Table S1). In some control experiments without DNA, DNase 1 was added at the start. When the Δ*uvrA* strain was used, ten assays (one ml per assay) were irradiated separately and pooled after irradiation of the cells. Cells were plated in appropriate dilution on LB with (survival transformant titer) and without kanamycin (10 mg l^−1^; survival recipient titer) and incubated for 24 h at 30 °C. Colonies were counted, the titers were determined, and the recipient and transformant survival frequencies were calculated as frequencies relative to those at UV_0_.

### Natural transformation experiments

The preparation of competent cells and the transformation assays were performed as described previously [58]. Briefly, the *A. baylyi* strains were grown in a shaker in LB broth at 30 °C to 1 × 10^9^ cells ml^−1^ (determined with a Neubauer hemocytometer) and stored at −80 °C as a concentrated stock (1 × 10^10^ cells ml^−1^ in LB with 10% glycerol) until use. For transformation assays, a freshly thawed bacterial suspension was diluted to 2.5 × 10^8^ cells ml^−1^ in LB containing 100 ng ml^−1^ donor DNA (isolated from *A. baylyi* KH12), aerated for two hours at 30 °C and plated in appropriate dilution on LB medium (recipient titer) and LB with 10 mg ml^−1^ kanamycin (transformants). The colonies were counted after 16 to 40 h at 30 °C. Transformation frequencies were calculated as transformants per recipient. No kanamycin-resistant colonies were obtained in control experiments without donor DNA.

### Statistical analysis

The relative fitness data were analysed using Two-ways ANOVA, with relative fitness as response variable and competitor strain (two levels), UV-exposure to cells (two levels) and DNA-availability (three levels) as explanatory variables. We also subjected the data to post hoc tests (with Tukey HSD correction) using the same model specifications. Confidence limits (95%) for the group means were obtained using formula based estimation.

The survival data were analysed by regression using a linear Mixed-model controlling for dependencies among the replicates from the same overnight culture used in a series of increasing UV-exposure. In our model, we used relative survival as response and UV-fluence (continuous) and total population or transformants (two levels) as explanatory variables, whereas the overnight culture was included as a random term. The observed trends were linearized by log transforming relative survival and the linearity of the trend was diagnosed, and confirmed, by generalized additive modelling [61]. The transformation frequencies of the set of recombinational repair deficient (Δ*recBCDFOR*) mutants were analysed by one-way ANOVA followed by post hoc tests with TukeyHSD correction. All analyses were performed with the statistical software R [62] and the additional R-packages lme and mgcv.

## Results

### Uptake of exogenous DNA is beneficial regardless of sequence homology, DNA damage and recombination

**Fig. 1:**
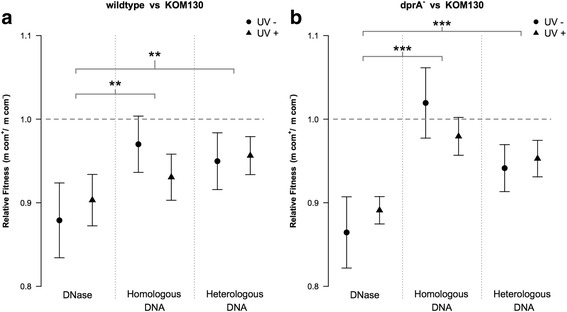
Relative fitness of wildtype strain (**a**) and its ∆*dprA* derivative (**b**) grown in head-to-head competitions against the DNA uptake-deficient ∆*comB-F* mutant. The competitions were treated with a combination of an initial UV pulse of 216 J m^−2^ or no UV irradiation with a subsequent amendment of no DNA (DNase), homologous DNA or heterologous DNA. In each group *n* = 13. In the control competitions with no UV and no DNA both the wildtype and its ∆*dprA* derivative displayed a reduced fitness relative to KOM130 of *w* = 0.88 (0.83–0.92, 95% CI) and *w* = 0.86 (0.82–0.91, 95% CI), respectively, due to the biological cost of type IV pilus expression (Additional file 1: Fig. S1). Error bars indicate 95% confidence intervals. Significant differences between the treatment groups (pooled results of both UV- and UV+ competitions) are indicated by asterisks (** *P* < 0.001, *** *P* < 0.0001). A two-way ANOVA of all mean values is provided in (Additional file 1: Table S4)

We investigated the general fitness effects of DNA uptake in mixed culture competition experiments with the transformation-proficient *A. baylyi* wildtype strain (LCQ2) and its non-transformable Δ*comB-F* derivative (Additional file 1: Table S1). The *comB-F* operon encodes genes required for type IV pilus formation, which is essential for transport of free DNA into the periplasm [20, 22, 56] and for spreading growth on semi-solid surfaces (Additional file 1: Fig. S1). To test for differences between benign and DNA damaging conditions, the mixed cultures were treated with or without initial pulses of UV irradiation, and were amended either with homologous DNA, heterologous DNA, or DNase. The UV-dose applied was 216 J m^−2^, which generates one photoproduct every 420 bp on average [63] and killed approximately 98% of the cells. In the absence of DNA, the wildtype strain displayed a relative fitness (*w*) reduction compared with the Δ*comB-F mutant* (Fig. 1a) [*w* = 0.88 (0.83–0.92, 95% CI)]. A mixed culture serial transfer experiment confirmed that the fitness reduction was due to the absence of type IV pilus components in the Δ*comB-F* competitor (Additional file 1: Fig. S2).

The addition of homologous or heterologous DNA increased the fitness of the wildtype compared to the DNA-free experiments (both *P* < 0.002) (Fig. 1a). The fitness effects of added DNA to the competitions were independent of UV-exposure (*P* = 0.83). Together these two results provide a strong argument in favour of a general benefit of DNA uncoupled from recombination.

To further investigate whether the observed fitness increase was independent of recombination, we used a DNA uptake-proficient but transformation-deficient mutant lacking the *dprA* gene [Additional file 1: Table S1] in head-to-head competitions with the Δ*comB-F* mutant. Absence of DprA abolished homologous recombination during natural transformation in *A. baylyi* as reported for *S. pneumoniae* [49] (detection limit 1 × 10^−9^ transformants/recipient). The Δ*dprA* mutant displayed the same cost of type IV pilus formation as the wildtype [*w* = 0.86 (0.82–0.91, 95% CI); Fig. S3]. As seen with the wildtype, addition of both heterologous and homologous DNA increased the fitness of the Δ*dprA* strain relative to the DNA uptake-deficient competitor Δ*comB-F* (both *P* < 0.001) regardless of UV-exposure (Fig. 1b). These data show that under the experimental conditions the DNA uptake machinery is costly to the cell. This cost can be partially compensated by addition of DNA to the competing cultures. However, the benefit of added DNA is unlinked to DNA recombination and thus DNA damage repair. This observation is further supported by the fact that *A. baylyi* takes up DNA from any source [44, 56], suggesting the benefit of competence is independent of DNA sequence homology.

### Homologous DNA reduces relative survival of transformants after UV-exposure

The competition experiments described above demonstrated a clear fitness increase for DNA-uptake-proficient cells regardless of the cells’ ability to use that DNA for recombination under benign or genotoxic conditions. However, the fitness benefit observed might obscure an additional benefit for the transformant fraction of cells in the culture, especially under DNA damaging conditions.

**Fig. 2:**
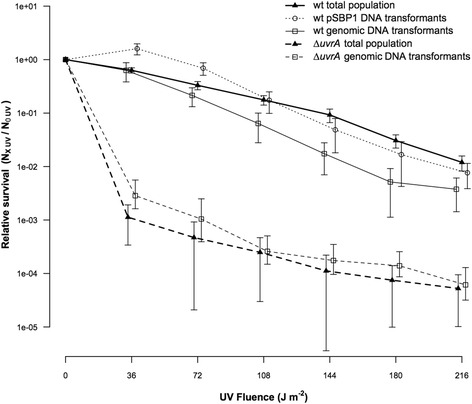
Relative survival of the pooled total population of the wildtype (closed triangles and bold solid line, *n* = 16) and the transformant fractions obtained with either pSBP1 DNA (0.1 μg ml^−1^; open circles with dotted line, *n* = 10) or with homologous genomic DNA (2 μg ml^−1^; open squares with solid line, *n* = 6) with increasing levels of UV irradiation. We also determined the relative survival of the total population (closed triangles and bold dashed line, *n* = 10) and the transformant fraction in the *∆uvrA* mutant obtained with homologous DNA (2 μg ml^−1^; open squares and dashed line, *n* = 10). Error bars denote the 95% confidence intervals. The transformation frequencies without UV irradiation were (7.7 ± 1.4) × 10^−4^ (pSBP1 DNA) and (2.5 ± 2.8) × 10^−2^ (genomic DNA) for the wildtype; and (1.9 ± 1.0) × 10^−2^ (genomic DNA) for the *∆uvrA* mutant. Mean initial titers (CFU/ml) for the wildtype and the *∆uvrA* mutant were 1.8 × 10^7^ and 4.2 × 10^7^, respectively

To test that, we investigated the effect of increasing intracellular UV damages in DNA on survival in the transformant fraction compared with the total population in monoculture experiments. We added a short donor DNA substrate (linearized pSBP1; [Additional file 1: Table S1]) containing a chromosomal *A. baylyi* segment (2 kbp) with a kanamycin resistance marker inserted in the center (*lifO-lipB*::*aphA3*) [22]. Single-locus homologous recombination is largely independent of the chromosomal position [64]. pSBP1 DNA can only be used for recombination at the *lifO-lipB* locus, and recombination results in kanamycin resistance. The donor DNA substrate was added in excess (0.1 μg ml^−1^), and the survival of transformants following UV irradiation from 0 to 216 J m^−2^ was quantified. The results showed an initial benefit of added pSBP1 DNA at low UV doses on survival of the transformant fraction (Fig. 2), similar to what was observed in *H. influenzae* [34]. However, at higher UV-doses (≥144 J m^−2^) that increased the likelihood of UV damages generated in the vicinity of the marker insertion site, this benefit was lost (Fig. 2). The result suggests that recombination with taken-up DNA interfered with repair of UV damages at the recombination locus, which is further supported by a steeper decline in survival of the transformant fraction with increasing UV-doses (Fig. 2 and [Additional file 1: Fig. S4 A]).

We hypothesized that fully homologous DNA as donor DNA would be even more disadvantageous because it can recombine and interfere with UV-damages genome-wide. We tested this by adding genomic *A. baylyi* donor DNA containing the *lifO-lipB*::*aphA3* marker (2 μg ml^−1^). The donor DNA substrate can undergo phenotypically silent recombinations at any chromosomal locus, and we tracked the integration of the *lifO-lipB*::*aphA3* marker as a proxy for the genome wide transformation frequency. Transformants survived less than the total population from low to high UV-doses (72–216 J m^−2^) (*P* < 0.001, Fig. 2 and [Additional file 1: Fig. S4 B]). The UV-survival curves of the total populations with pSBP1 or genomic DNA were indistinguishable and these data were pooled (Fig. 2). Control experiments without DNA revealed no difference in kill rates of the total population (Additional file 1: Fig. S4 D).

**Fig. 3:**
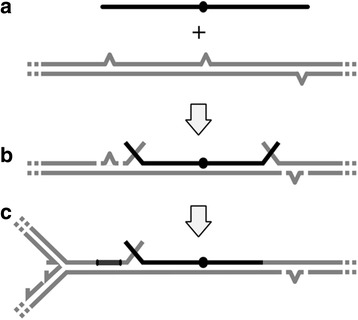
Formation of DNA double strand-breaks conferred by nucleotide excision repair during natural transformation. **a**: *Grey lines*: genomic DNA; grey spikes: UV-induced lesions. Black line: taken-up DNA single-strand with genetic marker (*oval*). **b**: Generation of DNA single-strand-breaks by UvrAB-directed, UvrBC-initiated cleavage of the damage-containing strand upstream and downstream of the lesions, and by RecA-mediated strand invasion of the taken-up DNA and cleavage of the displaced DNA strand. **c**: Partial repair of single-strand breaks: 1. by removal of the lesion-containing single-strand fragment (*left lesion*), fill-in synthesis, and covalent ligation (*dark grey*; catalysed by UvrD, DNA polymerase I, and DNA ligase, respectively); and 2. by covalent joining of the invaded taken-up strand with the genomic DNA at one side (downstream of the marker). Single-strand breaks persist when the UV lesion-containing strands are not removed (*right lesion*) and when an invaded DNA strand remains unligated at one end (upstream of the marker). One-sided ligation of the invaded DNA strand is common (see text). Following DNA replication (indicated by a replication fork approaching from the left end), single-strand breaks are converted into potentially lethal double-strand breaks

UV damages in bacteria can be repaired by nucleotide excision repair (NER) [65]. During NER, the UvrA-directed UvrBC excinuclease [59] cleaves one strand on either side of a UV- lesion in double-stranded DNA. Excision of the incised fragment with the lesion by helicase II is finally followed by fill-in synthesis by DNA polymerase I and sealing of the nick by DNA ligase. With increasing UV-doses, more NER repair sites would remain temporarily unfinished, and gaps and nicks would accumulate. In addition, recombination attempts with taken up DNA will add to the number of single strand breaks, as reported in [52]. We hypothesised that the decrease of transformant survival with increasing UV-doses was caused by interference of NER with transformational recombination (Fig. 3).

To test this hypothesis, we knocked out the key NER gene *uvrA* and investigated relative survival of transformants of the Δ*uvrA* strain with increasing UV-doses as outlined above. As expected, UvrA-deficiency resulted in greatly reduced survival when exposed to UV irradiation (Fig. 2). However, the Δ*uvrA* transformant fraction did not display any detrimental effect with increasing UV doses as observed in the wildtype (Fig. 2), and the kill rates of transformants in the absence of UvrA were the same as that of the total population (*P* = 0.79, [Additional file 1: Fig. S4 C]). This result suggests that in absence of NER, genome-wide recombination with homologous chromosomal DNA provided no additional burden, but also no benefit, to uptake-proficient cells. Cumulatively, NER and recombination with taken-up DNA become a burden to competent cells with increasing levels of DNA damages, due to generation of nicks and gaps that turn into DNA double-strand breaks when unrepaired. Consequently, successful integration of recombination attempts during natural transformation in the wildtype would depend on DNA strand-break repair functions.

### Genomic integration of taken-up DNA strongly depends on DNA repair

**Fig. 4:**
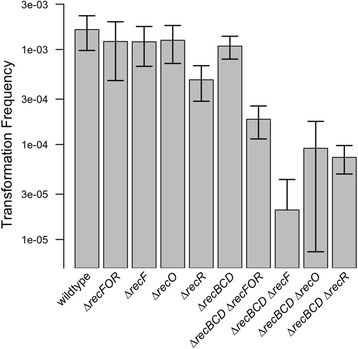
Natural transformation frequencies of *A. baylyi* wildtype and DNA recombination-impaired mutants. Transformation frequencies were obtained in liquid transformation experiments with genomic homologous DNA (0.1 μg ml^−1^) containing a kanamycin resistance marker gene and are given as means with 95% confidence intervals. The initial recipient titers are listed in (Additional file 1: Table S4)

Two studies on RecA-mediated recombination in *A. baylyi* [51, 52] suggested that both ends of a donor DNA molecule are integrated through two independent ligation events. While one end is covalently joined to the genomic DNA, the opposite end frequently remains temporally unligated, resulting in a single-strand break that can turn into a double-strand break following DNA replication. These reports [51, 52] are in agreement with the observed interaction between NER of UV-damages and recombination on transformant survival which suggest that incomplete recombination attempts during transformation may represent an additional source of DNA damages that rely on DNA strand break repair (Fig. 3). Recombinational DNA strand break repair is initiated in bacteria through two major pathways, the RecBCD and the RecFOR pathways of recombination [66, 67]. DNA single-strand breaks such as nicks or gaps can be repaired through the latter pathway, which requires among others the RecF, RecO and RecR gene products [68]. The RecFOR proteins in concert process gaps and generate RecA-loaded DNA 3′-ends for strand invasion and thus can initiate homologous reciprocal strand transfer and can also restore stalled and broken replication forks [68]. Alternatively, nicks and gaps can turn into double-strand breaks when unprocessed, and these damages are mainly repaired by the RecBCD pathway with the RecBCD heterotrimeric complex as the key repair function [69]. RecBCD processes DNA double-strand ends and generates a recombinogenic RecA-loaded 3′-end that initiates reciprocal strand exchange with a template for repair of the double-strand break [69]. To test the hypothesis that natural transformation depends on DNA strand break repair for successful integration of acquired DNA, we deleted components of the RecFOR pathway and the RecBCD pathway in *A. baylyi* and determined the effects of these deficiencies on natural transformation by homologous chromosomal DNA. The results are shown in Fig. 4. Deletions of *recF*, *recO* and *recR* in combination or alone overall had little or no effect on natural transformation frequencies compared with wildtype, as observed previously in *B. subtilis* and *S. pneumoniae* [70, 71], although in the Δ*recR* mutant it was decreased 3.3-fold (*P* = 0.007). Deletion of the *recBCD* operon, while highly detrimental to viability, did not affect transformation frequency (*P* = 0.94). A recent finding that RecR protein has a role in both RecBCD and RecFOR recombination pathways in mycobacteria [72] supports this observation. In contrast, in a Δ*recBCD* Δ*recFOR* mutant transformation was decreased approximately 10-fold (*P* < 0.001), and this decrease was strongly synergistic (about 5-fold higher than expected from the decreases in frequency of the single mutants, Fig. 4), confirming earlier reports on *H. pylori* [73]. This observation was confirmed using Δ*recF*, Δ*recO* or Δ*recR* mutants of the Δ*recBCD* strain (all *p*-values < 0.001; Fig. 4), with the Δ*recBCD* Δ*recF* double mutant yielding particularly low transformation frequencies (approximately 80-fold decreased compared with wildtype). It is conceivable that in this mutant, the functional RecOR proteins [74] generate recombination intermediates that cannot be resolved in the absence of RecF and RecBCD. An ANOVA table for multiple comparisons of means of the transformation frequencies is provided in (Additional file 1: Table S5). In summary, these results suggest that the majority of natural transformation events depends on DNA strand-break repair and support our hypothesis that taken-up DNA causes damages such as strand-breaks following recombination attempts. It can be hypothesized that amendment of DNA is detrimental to growing cells impaired in recombinational DNA repair. However, comparisons of serial transfer experiments of the Δ*recBCD* Δ*recFOR* mutant with and without DNA suggest that the hypothetical detrimental effect of added DNA on cell survival is masked by the initially observed beneficial effect of DNA (Fig. 1) on cell growth (Additional file 1: Fig. S5).

## Discussion

In this report we show that the costs of the DNA uptake machinery (*comB-F* gene products) in *A. baylyi* is offset by the amendment of DNA in competition experiments. The beneficial effect of added DNA was independent of UV-exposure to cells, DNA sequence homology, and unlinked to recombination. These results are inconsistent with a role of acquired DNA in strand-break repair as originally proposed by Bernstein and co-workers [30]. The high biological cost of type IV pilus formation (up to 14% reduction in relative fitness) suggests that the ability to take up DNA could rapidly be lost in naturally transformable populations. To this end, reduced transformability have been previously shown in *A. baylyi* during experimental evolution but this is not necessarily dependent on loss of DNA uptake functions since the cost of pilus formation appears to be medium-specific [20, 22]. It is also important to stress that the type IV pilus is involved in motility (Additional file 1: Fig. S1) suggesting that other selective forces may be responsible for the maintenance of central components of the DNA uptake machinery [75].

Early reports favoured the DNA repair hypothesis in *B. subtilis* [9, 31–33], and it has been demonstrated that *B. subtilis* displayed increased transformation frequencies during UV-exposure [33]. However, DNA damage does not induce the competence machinery in *B. subtilis* [35], and more recent reports have made a clear distinction between natural transformation and the physiological state of competence. For example, competence for natural transformation is induced by genotoxic stress in *S. pneumoniae* [38] and it was recently demonstrated that competence development following DNA-damaging stress was beneficial in the presence but not the absence of DNA in *S. pneumoniae* [36]. However, direct evidence demonstrating recombination between incoming donor DNA and damaged areas of the genome was not presented and it is not clear if this benefit was due to other processes, as also pointed out by the authors [36]. Interestingly, competence induction was also beneficial in the presence of non-DNA damaging stress, suggesting a general benefit that extends beyond recombination [36, 37]. Competence induction in *S. pneumoniae* involves quorum sensing of an extracellular competence-stimulating-peptide (CSP) for a short time during log phase and includes transcriptional regulation by ComX of high number of genes not only limited to DNA uptake and recombination [37]. This is consistent with our finding that taken up DNA increases relative fitness in *A. baylyi*. These results also provide support for a previous report on *H. influenzae*, where an increased survival of the whole population was demonstrated in the presence of both fully and partially homologous DNA [34]. Moreover, we show that the benefit of DNA is also present in an *A. baylyi* strain lacking a functional recombination mediator DprA. Since DprA loads taken-up single-stranded DNA with RecA, and the nucleoprotein filament subsequently initiates recombination [49], the result reported here strongly suggests that the fitness benefit of DNA uptake is not linked to recombination, but rather the uptake of free DNA.

The beneficial effect of DNA uptake does not necessarily exclude a role of the taken-up DNA for repair purposes in the recombining fraction of the competent population. We investigated the effect of exogenous DNA on transformant survival with increasing UV doses. The results revealed that recombination with taken-up DNA is costly. These data provide additional support for the proposed mechanistic model for, and consequences of, integration of DNA into the chromosome following uptake in *A. baylyi* [51, 52]. Kickstein and colleagues provided evidence in favour of separate integration of 5′- and 3′- donor DNA ends during recombination in *A. baylyi*, suggesting frequent occurrence of DNA single-strand breaks. If replication forks reach joints between donor and recipient DNA prior to ligation, a double-strand-break would occur [52]. This suggests that natural transformation in *A. baylyi* strongly depends on DNA strand break repair functions to ensure viability. In our DNA damage experiments we used UV-irradiation to induce intracellular DNA lesions that can lead to single- and double-strand breaks during DNA repair [76]. Consequently, the DNA repair machinery will be saturated due to multiple strand-breaks formed as a response to DNA damage. It is conceivable that the saturation affects initiation of recombinational repair (conferred by RecBCD or by RecFOR), but it is also possible that the repair processes exhaust the pool of cellular RecA protein which is required for initial strand invasion and for repair of strand-breaks due to incomplete insertions of the donor DNA. It can be hypothesized that the repair functions titrate RecA protein away from DprA and thus reduce transformation due to RecA shortage. However, it has been shown that DNA uptake in *A. baylyi* is initiated immediately after exposure to DNA [44], and in our UV experiments the cells were still in lag phase (before initiation of replication) when DNA was added. The integration of acquired DNA into the genome then is likely to cause additional stress in the recipient cells (Fig. 3).

Three lines of evidence presented in this report support this hypothesis. First, we demonstrate in UV-time-kill experiments that the transformant fraction is more susceptible to UV-killing than the total population. Second, UV-exposure reduced transformant survival due to interference between NER and natural transformation (Fig. 2). Third, under benign experimental conditions we demonstrate that the majority of natural transformation events depend on DNA repair functions as demonstrated in *recBCDFOR* mutants (Fig. 4). Taken together, the data presented here and previously [51, 52, 73] strongly suggest that recombination during natural transformation is costly and represents an additional hurdle that the selective forces responsible for the maintenance of natural transformation must overcome. Consequently, our findings are inconsistent with the DNA repair hypothesis for the evolution and maintenance of natural transformation in *A. baylyi* [9, 30].

If DNA uptake is beneficial and unlinked to recombination (which is detrimental), what causes this benefit? Taken-up DNA is rapidly degraded by single-strand-specific DNA exonucleases (RecJ and ExoX) in *A. baylyi* [56]. It is thus possible that the recipient cells can directly access this pool of nucleotides and incorporate them into the DNA biosynthesis pathways as suggested in previous reports [8, 77]. Our results would add to the recent finding that purine depletion induced the *H. influenzae* competence activator *sxy* [23], suggesting that DNA can be utilized as a nutrient or as building blocks in DNA metabolism.

## Conclusions

Our data suggest that the selective pressures responsible for the maintenance of natural transformation are not linked to recombination following DNA uptake. Our findings rather suggest that recombination is a DNA damaging process and that the positive effect of DNA uptake on *A. baylyi* fitness is due to free cytoplasmic DNA where it may be utilized as nutrition or as building blocks for DNA metabolism. In contrast, our data suggest that recombination during transformation is a strand break inducing process that represents a previously unrecognized cost of natural transformation. Our results add new insights to the costs and benefits that play a selective role in the maintenance of natural transformation.

## Additional files

Additional file 1: Supplementary Figures and Tables. (DOCX 1.33 mb)
Additional file 2: Supplementary Methods. (DOCX 13.6 kb)

## Abbreviations

bp: Base pairs
CI: Confidence interval
HSD: Honest significant difference
LB: Luria-Bertani
NER: Nucleotide excision repair
PBS: Phosphate-buffered saline
UV: Ultraviolet
