# Supplementary material for: Costs and benefits of natural transformation in Acinetobacter baylyi
Source: BMC Microbiol. 2017 Feb 15;17:34. doi: 10.1186/s12866-017-0953-2 (PMC5312590; doi:10.1186/s12866-017-0953-2)
Supplement: Additional file 1: — Supplementary Figures and Tables. (DOCX 1.33 mb) [file 12866_2017_953_MOESM1_ESM.docx]

**Supplementary Figures and Tables**

**Cost and benefits of natural transformation in *Acinetobacter baylyi***

Nils Hülter, Vidar Sørum, Kristina Borch-Pedersen, Mikkel M. Liljegren, Ane L.G. Utnes, Raul Primicerio, Klaus Harms and Pål J. Johnsen

KOM130 LCQ2


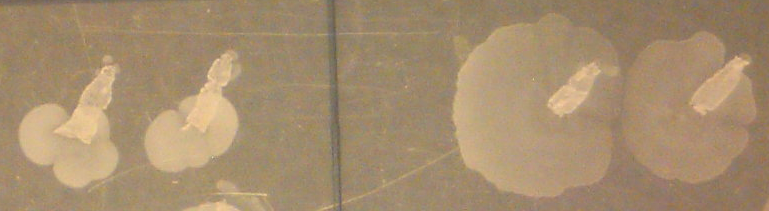


**Figure S1:** Spreading growth phenotypes of DNA uptake-deficient (KOM130; Δ*comB-F*::*dhfr1*) and -proficient (LCQ2; wildtype) *A. baylyi* strains confirming that spreading growth is facilitated by type IV pili. Plates with semi-solid (0.4% agar) LB medium were inoculated with a straight wire containing fresh cellular material and by stabbing through the agar to the bottom of the Petri dish. Spreading growth of cells between the Petri dish bottom surface and the semi-solid agar after incubation for 16 hours at 30°C was impaired in the absence of the *comB-F* gene products encoding type IV pilus components, confirming Type IV-pilus deficiency.

**Figure S2**: The biological cost of the type IV pilus expression in *A. baylyi* was determined in serial transfer competition experiments. Changes in fitness of a tested strain compared with a competitor strain are measured by tracking changes in LN ratios over time and are displayed as symbols with regression lines. Serial competition experiments were performed as published [1]. Cross symbols and solid line: wildtype competed against the ∆*comB-F*::*dhfr1* (trimethoprim-resistant) strain using trimethoprim resistance as selective marker. The negative slope indicates the biological cost. Open circles and dashed line: We competed wildtype against the ∆*comB-F*::*nptII* (kanamycin-resistant) mutant and confirmed the fitness decrease of the first experiment with a different and previously confirmed fitness neutral selection regime [2] (kanamycin resistance). The ∆*comB-F*::*nptII* substitution mutation was constructed using the plasmid pGT41 as described in the materials and methods section in the main text. Filled circles and dashed line: ∆*comB-F*::*dhfr1* competed against ∆*comB-F*::*nptII* using trimethoprim resistance as selective marker. The neutral slope indicates the absence of any fitness cost of the selective marker. The average relative fitness between the competitiors as estimated from the slopes were as follows: *w* = 0.87 for wildtype/∆*comB-F*::*dhfr1*; *w* = 0.85 for wildtype/∆*comB-F*::*nptII;* and *w* = 1.00 for ∆*comB-F*::*dhfr1*/∆*comB-F*::*nptII*. These results confirm the relative fitness advantage in type IV pilus-deficient competitors observed in our competition experiments (Fig. 1) and the neutrality of the selective marker, respectively.

**Figure S3**: Relative fitness of NH29 (Δ*dprA trpE*^+^) compared to JV28 (*dprA*^+^ *trpE^-^*). Relative fitness above one would indicate a fitness benefit to NH29. The measured fitness was not significantly different from one [*w* = 1.014 (0.99-1.39, 95% CI), *P* = 0.22, n = 6; One sample t-test]. Relative fitness was estimated from the slopes of serial transfer competitions (4 passages) performed as published [1] The competitors were scored by selective plating on LB and on minimal M9 medium supplemented with succinate.

**Figure S4:** Relative survival (CFU_X UV_ / CFU_No_ _UV_) of the total population (closed circles, solid line) and the transformant fraction (open circles, dashed line) with increasing levels of UV-fluence (joule m^-2^). The headings indicate the donor DNA substrate, and the genotype employed in the experiments (wildtype unless indicated otherwise). **A**: When wildtype cells were exposed to pSBP1 DNA, we observed an increased survival of transformants at low UV doses but decreased survival at high doses (difference in slope: *β =*-0.009, t-value=-5.34, *P*<0.001; difference in intercept: *β =* 1.146, t-value= 4.959, *P*<0.001). **B**: When homologous genomic DNA was used, the relative survival of the transformant fraction compared with the total population was decreased with increasing UV-fluence. The decrease rate was similar to that observed with pSBP1 (difference in slope: *β =*-0.011, t-value=-5.49, *P*<0.001), but survival was lower throughout (difference in intercept: *β =* -0.016, t-value=- 0.057, *P*= 0.95). **C**: In the Δ*uvrA* mutant, the survival of the transformant fraction did not differ from that of the total population (difference in slope: *β =*0.001, t-value=0.267, *P*=0.78; difference in intercept: *β =* 0.039, t-value=0.093, *P*= 0.93). **D**: The survival of the total population in absence of DNA showed a decrease in survival similar to that of the total population supplemented with genomic DNA (grey dashed line).

**Figure S5:** Overnight cultures of the *ΔrecBCD ΔrecFOR* strain wore diluted 100-fold, exposed to either isogenic donor DNA or DNase and propagated for two hours in LB broth for altogether ten iterations (two-fold dilutions). Viable cell counts were determined for each treatment after one, five and ten rounds of exposure. We did not find any difference in viable cell counts between the DNA and DNase treatment after one, five and ten iterations, p-values > 0.2 estimated using ANOVA with Tukey HSD correction for multiple comparisons of means. *N* = 10 for both treatments. We observed a weak trend (non-significant) towards higher colony counts in the experiments with DNA which we attribute to the beneficial effect of donor DNA on transformable cells (see main manuscript). It is likely that the demonstrated beneficial effect of exogenous DNA masks the relatively small effect of additional strand breaks by DNA invasion and incomplete integration.

**Table S1**: List of *A. baylyi* strains and donor DNA substrates.

| ***Strain or DNA substrate*** | ***Genotype*** | ***Reference*** |
| --- | --- | --- |
| ***Strain*** |  |  |
| JV28 | ADP1 *trpE27 rpoB1(*Rif^r^) *alkM*::(*nptII*’ *tg4*) | [3] |
| LCQ2 | JV28 trpE^+^ ("wildtype") | This study |
| KOM130 | JV28 ∆*comB-F*::*dhfr1* (Tm^R^) | This study |
| NH33 | JV28 ∆ *comB-F*::*nptII* (Km^R^) | This study |
| NH29 | LCQ2 *∆dprA*::*aacC1* (Gm^R^) | This study |
| KOM141 | JV28 ∆uvrA | This study |
| MKD3 | JV28 ∆recFOR | This study |
| MKD1 | JV28 ∆recF | This study |
| KOM82 | JV28 ∆recO | This study |
| MKD2 | JV28 ∆recR | This study |
| KOM18 | JV28 ∆recBCD | [4] |
| MKD6 | JV28 ∆recBCD recFOR | This study |
| MKD4 | JV28 ∆recBCD recF | This study |
| KOM86 | JV28 ∆recBCD recO | This study |
| MKD5 | JV28 ∆recBCD recR | This study |
| ***DNA substrate*** |  |  |
| ADP1200Com+^KanR^ | ADP1 *lifO-lipB*::*aphA3* (Km^R^) | [5] |
| KH12 | ADP1 *alkM*::(*nptII*^+^ *tg4*) (Km^R^) | This study |
| pSBP1 | pUC19 with *lifO-lipB*::*aphA3** (Km^R^)  kbp) | This study |
| Salmon sperm DNA | heterologous DNA substrate | Sigma-Aldrich |

*trpE^+^* – tryptophan prototrophic; Tm^R^ – trimethoprim resistant; Kan^R^ – kanamycin resistant; Gm^R^ – gentamicin resistant.

*total size: 5.2 kbp, including a 2.0 kb segment homologous to the *A. baylyi* chromosome with a central *aphA3* (0.5 kbp) insertion.

**Table S2:** List of plasmids.

| Name | Relevant genotype | Function | Phenotype |
| --- | --- | --- | --- |
| pKH119 | pGT41 Δ*uvrA*::(*nptII sacB*) | substitution of *uvrA* | Cm^R^ Ap^R^ Km^R^ Sucr^S^ |
| pKH119 | pGT41 Δ*uvrA* | deletion of *uvrA* | Cm^R^ Ap^R^ |
| pLNSS2 | pGT41 Δ*recF*::(*nptII sacB*) | substitution of *recF* | Cm^R^ Ap^R^ Km^R^ Sucr^S^ |
| pLNSS3 | pGT41 Δ*recF* | deletion of *recF* | Cm^R^ Ap^R^ |
| pLNSS5 | pGT41 Δ*recR*::(*nptII sacB*) | substitution of *recR* | Cm^R^ Ap^R^ Km^R^ Sucr^S^ |
| pLNSS6 | pGT41 Δ*recR* | deletion of *recR* | Cm^R^ Ap^R^ |
| pBlue-recO11 | pGT33 Δ*recO*::(*nptII sacB*) | substitution of *recO* | Ap^R^ Km^R^ Sucr^S^ |
| pBlue-recO10 | pGT33 Δ*recO* | deletion of *recO* | Ap^R^ |
| pKH83 | pGT41 Δ*recBCD*::(*nptII sacB*) | substitution of *recBCD* | Cm^R^ Ap^R^ Km^R^ Sucr^S^ |
| pKH84 | pGT41 Δ*recBCD* | deletion of *recBCD* | Cm^R^ Ap^R^ |
| pSBP1 | pUC19 *lifO-lipB*::*aphA3* | Km^R^ donor DNA | Ap^R^ Km^R^ |

Cm^R^ – chloramphenicol resistance; Ap^R^ – ampicillin resistance; Km^R^ – kanamycin resistance; Sucr^S^ – sucrose susceptibility

**Table S3:** List of primers.

| Name | DNA sequence (5'-3') |
| --- | --- |
| dprA_1 | TCAATCTGTCCCACATCACCAG |
| dprA_2 | GCCCTAGGCTCCCATACCACGCGCGCCTTGAAACAGCACATACGAG |
| dprA_5 | CCGCTAGCAATTACCTACCGGCGTTCTTTTCGAATTGCTGCTGTCC |
| dprA_6 | AGGTGTATGTTCGTGGTCAAGG |
| aacC1_3 | GCGCGTGGTATGGGAGCCTAGGGCTGTTAGGTGGCGGTACTTGG |
| aacC1_4 | ACGCCGGTAGGTAATTGCTAGCGGGGC**T**T**GAC**AGCAGCAAGCGCGTTA**TAAT**GTGGGTCG |
| uvrA-up-f | GTAAACGGTTGGCAAAGTAATAC |
| uvrA-up-r | AGAGCTCTGTTCAAGCACAGGCTTTGG |
| uvrA-down-f | CTTCTAGAGCTCACATACTGGGCGTTTCTTAAAGC |
| uvrA-down-r | AACTTGATTTTGCCAAAAAATAGAACC |
| recF-up-f | GTAAGGGGCATGCATTGAAGCTGG |
| recF-up-r | ATCTAGACACACGCATTGGCATGACC |
| recF-down-f | CTTCTAGACGTTGATTTTTTACCCATCTTTGG |
| recF-down-r | AACTTGATAACTATTTAAACCACGTGTC |
| recR-up-f | GTAAACTTTACGTTTAAGAATGAAAGC |
| recR-up-r | ATCTAGACTAAACACGTTATCTTTATCC |
| recR-down-f | CTTCTAGAAAATGAAATAGACAATTTGG |
| recR-down-r | AACTTGATAACTGGAATGTATTTCTAGG |
| ACIAD3308-up-f | GTAAGTCTTCCCCAGCCTGCACG |
| ACIAD3309-down-r | AACTTGAGCGTCTTCAAGCATTTGAAGG |
| comF-up-fw | GTAATCTACGCCCCAATTGAC |
| comF-up-rv | ATCTAGAGACGTAGAATATCGCCCTCTATGAAAGG |
| comB-down-fw_3 | CTTCTAGATTAGTACGCCTCCAGAAACAAACACG |
| comB-down-rv | AACTTGAACAGCAATGGTTTGATGGTGTGGC |

Overlapping 5’-extensions of primers used in SOE-PCR are underlined. Primer aacC1_4 introduced nucleotide exchanges (bold) upstream of *aacC1*, generating a modified CP6 promoter [6, 7] replacing the native, distantly located *aacC1* promoter of Tn*1996* [8].

**Table S4:** ANOVA with Tukey HSD correction for multiple comparisons of means of relative fitness in competitions between the transformation proficient wildtype and the uptake-deficient Δ*comB-F* strain (KOM130), and the uptake-proficient but transformation-deficient Δ*dprA* against Δ*comB-F*. See Materials and Methods section for model specification.

|  | **Mean** | **95 % Confidence Interval** | |  |
| --- | --- | --- | --- | --- |
| **Pairwise comparison** | **difference** | **lower bound** | **upper bound** | **p adjusted** |
| **DNA** |  |  |  |  |
| heterologous DNA vs DNase | 0.066 | 0.041 | 0.090 | < 0.001 |
| homologous DNA vs DNase | 0.090 | 0.066 | 0.115 | < 0.001 |
| homologous DNA vs heterologous DNA | 0.025 | 0.000 | 0.049 | 0.046 |
| **Strain** |  |  |  |  |
| wildtype vs Δ*dprA* | -0.010 | -0.027 | 0.007 | 0.238 |
| **UV-exposure** |  |  |  |  |
| UV vs noUV | -0.002 | -0.018 | 0.015 | 0.833 |
| **DNA*UV-exposure** |  |  |  |  |
| heterologous DNA & noUV vs DNase & noUV | 0.074 | 0.032 | 0.116 | < 0.001 |
| homologous DNA & noUV vs DNase & noUV | 0.123 | 0.081 | 0.165 | < 0.001 |
| DNase & UV vs DNase & noUV | 0.025 | -0.017 | 0.067 | 0.508 |
| heterologous DNA & UV vs DNase & noUV | 0.083 | 0.041 | 0.125 | < 0.001 |
| homologous DNA & UV vs DNase & noUV | 0.083 | 0.041 | 0.125 | < 0.001 |
| homologous DNA & noUV vs heterologous DNA & noUV | 0.049 | 0.007 | 0.091 | 0.012 |
| DNase & UV vs heterologous DNA & noUV | -0.049 | -0.091 | -0.006 | 0.014 |
| heterologous DNA & UV vs heterologous DNA & noUV | 0.009 | -0.033 | 0.051 | 0.989 |
| homologous DNA & UV vs heterologous DNA & noUV | 0.009 | -0.033 | 0.052 | 0.987 |
| DNase & UV vs homologous DNA & noUV | -0.098 | -0.140 | -0.056 | < 0.001 |
| heterologous DNA & UV vs homologous DNA & noUV | -0.040 | -0.082 | 0.002 | 0.072 |
| homologous DNA & UV vs homologous DNA & noUV | -0.040 | -0.082 | 0.002 | 0.077 |
| heterologous DNA & UV vs DNase & UV | 0.058 | 0.015 | 0.100 | 0.002 |
| homologous DNA & UV vs DNase & UV | 0.058 | 0.016 | 0.100 | 0.002 |
| homologous DNA & UV vs heterologous DNA & UV | 0.000 | -0.042 | 0.042 | 1.000 |
| **DNA*Strain** |  |  |  |  |
| heterologous DNA & Δ*dprA* vs DNase & Δ*dprA* | 0.069 | 0.027 | 0.111 | < 0.001 |
| homologous DNA & Δ*dprA* vs DNase & Δ*dprA* | 0.122 | 0.080 | 0.164 | < 0.001 |
| DNase & wildtype vs DNase & Δ*dprA* | 0.013 | -0.029 | 0.055 | 0.942 |
| heterologous DNA & wildtype vs DNase & Δ*dprA* | 0.075 | 0.033 | 0.117 | < 0.001 |
| homologous DNA & wildtype vs DNase & Δ*dprA* | 0.073 | 0.030 | 0.115 | < 0.001 |
| homologous DNA & Δ*dprA* vs heterologous DNA & Δ*dprA* | 0.052 | 0.010 | 0.094 | 0.006 |
| DNase & wildtype vs heterologous DNA & Δ*dprA* | -0.056 | -0.098 | -0.014 | 0.002 |
| heterologous DNA & wildtype vs heterologous DNA & Δ*dprA* | 0.006 | -0.036 | 0.048 | 0.999 |
| homologous DNA & wildtype vs heterologous DNA & Δ*dprA* | 0.003 | -0.039 | 0.045 | 1.000 |
| DNase & wildtype vs homologous DNA & Δ*dprA* | -0.108 | -0.150 | -0.066 | < 0.001 |
| heterologous DNA & wildtype vs homologous DNA & Δ*dprA* | -0.046 | -0.088 | -0.004 | 0.022 |
| homologous DNA & wildtype vs homologous DNA & Δ*dprA* | -0.049 | -0.091 | -0.007 | 0.012 |
| heterologous DNA & wildtype vs DNase & wildtype | 0.062 | 0.020 | 0.104 | < 0.001 |
| homologous DNA & wildtype vs DNase & wildtype | 0.059 | 0.017 | 0.101 | 0.001 |
| homologous DNA & wildtype vs heterologous DNA & wildtype | -0.003 | -0.045 | 0.039 | 1.000 |

**Table S5:** ANOVA with Tukey HSD correction for multiple comparisons of means of transformation frequencies of wildtype and DNA recombination-impaired *A. baylyi* strains. See Materials and Methods section for model specification.

|  | **Mean** | **95 % Confidence Interval** | |  |
| --- | --- | --- | --- | --- |
| **Pairwise comparison** | **difference** | **lower bound** | **upper bound** | **p adjusted** |
| Δ*recBCD* Δ*recF* - Δ*recBCD* | -4.0916 | -5.143 | -3.040 | < 0.001 |
| Δ*recBCD* Δ*recFOR* - Δ*recBCD* | -1.839 | -2.626 | -1.052 | < 0.001 |
| Δ*recBCD* Δ*recO* - Δ*recBCD* | -2.691 | -3.743 | -1.640 | < 0.001 |
| Δ*recBCD* Δ*recR* - Δ*recBCD* | -2.697 | -3.506 | -1.887 | < 0.001 |
| Δ*recF* - Δ*recBCD* | 0.110 | -0.861 | 1.081 | >0.999 |
| Δ*recFOR -*Δ*recBCD* | 0.036 | -0.877 | 0.950 | 1.000 |
| Δ*recO* - Δ*recBCD* | 0.169 | -0.801 | 1.140 | >0.999 |
| Δ*recR* - Δ*recBCD* | -0.800 | -1.714 | 0.113 | 0.135 |
| Wildtype - Δ*recBCD* | 0.325 | -0.461 | 1.111 | 0.935 |
| Δ*recBCD* Δ*recFOR* - Δ*recBCD* Δ*recF* | 2.252 | 1.187 | 3.317 | < 0.001 |
| Δ*recBCD* Δ*recO* - Δ*recBCD* Δ*recF* | 1.399 | 0.126 | 2.673 | 0.020 |
| Δ*recBCD* Δ*recR* - Δ*recBCD* Δ*recF* | 1.394 | 0.312 | 2.476 | 0.003 |
| Δ*recF* - Δ*recBCD* Δ*recF* | 4.201 | 2.993 | 5.409 | < 0.001 |
| Δ*recFOR* - Δ*recBCD* Δ*recF* | 4.128 | 2.965 | 5.290 | < 0.001 |
| Δ*recO* - Δ*recBCD* Δ*recF* | 4.261 | 3.053 | 5.469 | < 0.001 |
| Δ*recR* - Δ*recBCD* Δ*recF* | 3.291 | 2.128 | 4.453 | < 0.001 |
| wildtype - Δ*recBCD* Δ*recF* | 4.416 | 3.351 | 5.482 | < 0.001 |
| Δ*recBCD* Δ*recO* - Δ*recBCD* Δ*recFOR* | -0.852 | -1.917 | 0.212 | 0.227 |
| Δ*recBCD* Δ*recR* - Δ*recBCD* Δ*recFOR* | -0.857 | -1.685 | -0.030 | 0.036 |
| Δ*recF* - Δ*recBCD* Δ*recFOR* | 1.949 | 0.963 | 2.935 | < 0.001 |
| Δ*recFOR* - Δ*recBCD* Δ*recFOR* | 1.875 | 0.945 | 2.805 | < 0.001 |
| Δ*recO* - Δ*recBCD* Δ*recFOR* | 2.008 | 1.022 | 2.995 | < 0.001 |
| Δ*recR* - Δ*recBCD* Δ*recFOR* | 1.038 | 0.109 | 1.968 | 0.017 |
| wildtype - Δ*recBCD* Δ*recFOR* | 2.164 | 1.359 | 2.969 | < 0.001 |
| Δ*recBCD* Δ*recR* - Δ*recBCD* Δ*recO* | -0.005 | -1.087 | 1.076 | 1.000 |
| Δ*recF* - Δ*recBCD* Δ*recO* | 2.801 | 1.593 | 4.009 | < 0.001 |
| Δ*recFOR* - Δ*recBCD* Δ*recO* | 2.728 | 1.565 | 3.890 | < 0.001 |
| Δ*recO* - Δ*recBCD* Δ*recO* | 2.861 | 1.653 | 4.069 | < 0.001 |
| Δ*recR* - Δ*recBCD* Δ*recO* | 1.891 | 0.729 | 3.053 | < 0.001 |
| wildtype - Δ*recBCD* Δ*recO* | 3.016 | 1.951 | 4.082 | < 0.001 |
| Δ*recF* - Δ*recBCD* Δ*recR* | 2.807 | 1.802 | 3.811 | < 0.001 |
| Δ*recFOR* - Δ*recBCD* Δ*recR* | 2.733 | 1.784 | 3.682 | < 0.001 |
| Δ*recO* - Δ*recBCD* Δ*recR* | 2.866 | 1.862 | 3.871 | < 0.001 |
| Δ*recR* - Δ*recBCD* Δ*recR* | 1.896 | 0.947 | 2.846 | < 0.001 |
| wildtype - Δ*recBCD* Δ*recR* | 3.022 | 2.194 | 3.849 | < 0.001 |
| Δ*recFOR* - Δ*recF* | -0.073 | -1.163 | 1.016 | 1.000 |
| Δ*recO* - Δ*recF* | 0.059 | -1.079 | 1.198 | 1.000 |
| Δ*recR* - Δ*recF* | -0.910 | -2.000 | 0.180 | 0.180 |
| wildtype - Δ*recF* | 0.214 | -0.771 | 1.201 | >0.999 |
| Δ*recO* - Δ*recFOR* | 0.133 | -0.957 | 1.223 | >0.999 |
| Δ*recR* - Δ*recFOR* | -0.836 | -1.876 | 0.202 | 0.220 |
| wildtype - Δ*recFOR* | 0.288 | -0.641 | 1.218 | 0.989 |
| Δ*recR* - Δ*recO* | -0.969 | -2.060 | 0.120 | 0.122 |
| wildtype - Δ*recO* | 0.155 | -0.830 | 1.141 | >0.999 |
| wildtype - Δ*recR* | 1.125 | 0.195 | 2.055 | 0.006 |

**Table S6:** Transformation frequencies for Δ*recBCDFOR* experiments presented in Fig. 3.

| **Strain** | **Genotype** | **Recipient live titer [ml^-1^]** |
| --- | --- | --- |
| JV28 | "wildtype" | (2.3±0.8)×10^8^ |
| MKD1 | Δ*recF* | (2.3±0.9)×10^8^ |
| KOM82 | Δ*recO* | (5.1±3.6)×10^7^ |
| MKD2 | Δ*recR* | (4.4±0.4)×10^7^ |
| MKD3 | Δ*recFOR* | (1.6±0.7)×10^8^ |
| KOM18 | Δ*recBCD* | (1.6±0.2)×10^8^ |
| MKD6 | Δ*recBCD* Δ*recFOR* | (2.4±0.7)×10^7^ |
| MKD4 | Δ*recBCD* Δ*recF* | (8.7±3.9)×10^6^ |
| KOM86 | Δ*recBCD* Δ*recO* | (7.8±12.1)×10^7^ |
| MKD5 | Δ*recBCD* Δ*recR* | (2.3±0.6)×10^8^ |

**Supplementary References**

[1] Starikova, I., Al-Haroni, M., Werner, G., Roberts, A.P., Sorum, V., Nielsen, K.M. & Johnsen, P.J. 2013 Fitness costs of various mobile genetic elements in Enterococcus faecium and Enterococcus faecalis. *J Antimicrob Chemother* **68**, 2755-2765. (doi:10.1093/jac/dkt270).

[2] Starikova, I., Harms, K., Haugen, P., Lunde, T.T.M., Primicerio, R., Samuelsen, O., Nielsen, K.M. & Johnsen, P.J. 2012 A Trade-off between the Fitness Cost of Functional Integrases and Long-term Stability of Integrons. *PLoS Pathog* **8**. (doi:ARTN e1003043

10.1371/journal.ppat.1003043).

[3] de Vries, J., Heine, M., Harms, K. & Wackernagel, W. 2003 Spread of recombinant DNA by roots and pollen of transgenic potato plants, identified by highly specific biomonitoring using natural transformation of an Acinetobacter sp. *Appl Environ Microbiol* **69**, 4455-4462.

[4] Kickstein, E., Harms, K. & Wackernagel, W. 2007 Deletions of recBCD or recD influence genetic transformation differently and are lethal together with a recJ deletion in Acinetobacter baylyi. *Microbiology* **153**, 2259-2270. (doi:10.1099/mic.0.2007/005256-0).

[5] Utnes, A.L., Sorum, V., Hulter, N., Primicerio, R., Hegstad, J., Kloos, J., Nielsen, K.M. & Johnsen, P.J. 2015 Growth phase-specific evolutionary benefits of natural transformation in Acinetobacter baylyi. *The ISME journal* **9**, 2221-2231. (doi:10.1038/ismej.2015.35).

[6] Jensen, P.R. & Hammer, K. 1998 Artificial promoters for metabolic optimization. *Biotechnol Bioeng* **58**, 191-195.

[7] Poteete, A.R., Rosadini, C. & St Pierre, C. 2006 Gentamicin and other cassettes for chromosomal gene replacement in Escherichia coli. *Biotechniques* **41**, 261-262, 264.

[8] Wohlleben, W., Arnold, W., Bissonnette, L., Pelletier, A., Tanguay, A., Roy, P.H., Gamboa, G.C., Barry, G.F., Aubert, E., Davies, J., et al. 1989 On the evolution of Tn21-like multiresistance transposons: sequence analysis of the gene (aacC1) for gentamicin acetyltransferase-3-I(AAC(3)-I), another member of the Tn21-based expression cassette. *Mol Gen Genet* **217**, 202-208.
