# Supplementary material for: Costs and benefits of natural transformation in Acinetobacter baylyi
Source: BMC Microbiol. 2017 Feb 15;17:34. doi: 10.1186/s12866-017-0953-2 (PMC5312590; doi:10.1186/s12866-017-0953-2)
Supplement: Additional file 2: — Supplementary Methods. (DOCX 13.6 kb) [file 12866_2017_953_MOESM2_ESM.docx]

**Supplementary Methods**

*Construction of the A. baylyi ΔdprA allele*

Splicing by Overlap Extension PCR: Two primary PCR reactions were performed to amplify chromosomal DNA segments of *A. baylyi* upstream and downstream of the desired insertion site in *dprA* (upstream segment A: 837 bp amplified with primers dprA_1/dprA_2; downstream segment C: 851 bp, amplified with dprA_5/dprA_6; all primer sequences are listed in [Additional file 1, Table S3]. A third primary PCR fragment (fragment B) containing the *aacC1* gene with promoter [646-bp using aacC1_3/aacC1_4 with plasmid pUC18T-miniTn7T-Gm-eyfp (GenBank: DQ493879) as template] was amplified with primers that carried overlaps with the PCR products A and C at both ends. PCR products A, B and C were used as template in a secondary PCR reaction to generate a linear fragment (ABC) containing the Δ*dprA*::*aacC1* allele. DreamTaq polymerase (Thermo Scientific; Waltham, MA) was used according to the manufacturer's guidelines in 20 μl reaction volumes. For the secondary reaction, one μl of each unpurified primary PCR product was used as template in a 50 μl PCR reaction volume and amplified with dprA_1/dprA_6. The resulting PCR product was used directly for natural transformation of strain LCQ2 [Additional file 1, Table S1]. Transformants were scored on LB plates supplemented with gentamicin (4 μg ml^-1^). The Δ*dprA*::*aacC1* mutation in the resulting strain NH29 was confirmed by PCR. The mutation rendered NH29 non-transformable (detection limit: 10^-9^).

*Construction of the A. baylyi ΔuvrA, ΔrecF and ΔrecR alleles*

Using *uvrA* as example, two fragments of approximately one kb each upstream and downstream of the *A. baylyi uvrA* gene were PCR-amplified separately using primers uvrA-up-f/uvrA-up-r or uvrA-down-f/uvrA-down-r and sequentially inserted into the *Oli*I and *Ksp*AI sites of the cloning vector pGT41, generating a Δ*uvrA*::(*nptII sacB*) substitution allele (conferring kanamycin resistance and sucrose susceptibility). From the resulting plasmid, the *nptII sacB* genes were excised with *Xba*I, resulting in a plasmid containing the Δ*uvrA* allele (sucrose-resistant, kanamycin-sensitive) embedded in its natural flanking sequences. The *recF* and *recR* deletion alleles were constructed accordingly with the respective primer pairs [Additional file 1, Table S3] and pGT41 as cloning vector.
